# Authentic gender development in non-binary children

**DOI:** 10.3389/fsoc.2023.1177766

**Published:** 2023-06-20

**Authors:** Fernando Salinas-Quiroz, Noah Sweder

**Affiliations:** “Abby and Anna” Sexual Orientation, Gender Identity and Expression (SOGIE) Lab, Eliot-Pearson Department of Child Study and Human Development (EPCSHD), School of Arts and Sciences, Tufts University, Medford, MA, United States

**Keywords:** gender development, gender identity, non-binary children, early childhood, socialization, child development, child studies

## Abstract

At present, the conceptualization of gender as a spectrum as well as non-binary identities have become increasingly visible and embraced. We are using non-binary as an umbrella term that refers to individuals who self-identify as a gender outside the gender binary, and/or who do not identify as always and completely being just a man or a woman. Our goal is to begin to create a framework for understanding gender development in non-binary children ages 0 to 8, since previous models have operated on cissupremacist assumptions, not applicable to non-binary people. As there is virtually no empirical data on the subject, we conducted a thorough literature review of current gender development theories and used our positionality as non-binary researchers to postulate two minimum criteria for non-binary gender identification: that a child learns about the existence of non-binary identities, and that they do not identify with the definitions they have been taught of what a boy or girl is. Children can learn about non-binary identities through media and knowledgeable community members and can develop “gender traits” authentically and come to identify as non-binary through biological predispositions, parental support, modeling, and being in peer groups that are supportive of identity exploration. Yet, children are not simply a product of their nature and nurture, as evidence has shown that humans are active agents in their gender development from a young age.

## 1. Introduction

The modern gender binary is not a natural human tendency, as it is made out to be. According to Bederman (1995), in the early 20^th^ century, eugenic scientists and policy makers used the rhetoric of “civilization” to naturalize white supremacy and patriarchy. They argued that the United States (US) was destined to become the pinnacle of racial evolution. Policing sex became seen as necessary to advance the white race and stave off the threat of racial decay, i.e., “primitive” gender non-conformity. American masculinity became redefined as a form of racial genius that was only achievable by white people and inaccessible by “savages” who were not seen as advanced enough to display sex differences between men and women. It is undeniable that racism is foundational to gender norms and gender norms are essential to racism (Bederman, 1995). Owing to Black and Brown trans resistance and activism, many people in contemporary societies have re-opened to the idea that gender is a spectrum. Nevertheless, research about gender development has almost exclusively focused on cisgender boys and girls. While in the past couple of years some research has been published about gender development in trans youth (e.g., Gülgöz et al., 2019; Olson et al., 2022), it has focused on trans boys and girls, and not on non-binary children. Moreover, the authors have continued to use concepts of gender development theories that were conceived by and for cisgender individuals that cannot be automatically extended to non-binary people. While trans is an umbrella term for people who do not identify with the gender they were assigned at birth, we will be exclusively focusing on gender development in non-binary people. As there are countless identities that do not fit into the binary, for the purposes of this paper we are using non-binary as an umbrella term for someone who self-identifies as a gender outside the gender binary, and/or does not identify as always and completely being just a boy/man or a girl/woman. Some examples of non-binary identities include: being both a boy and a girl, genderfluid and fluctuating in gender identity, demigender (relating more as one gender than another, but not as completely that gender), bigender (having two gender identities), trigender (having three gender identities), two-spirit, another third gender according to a cultural heritage, xenogender (identifying with a thing or concept seemingly unrelated to gender), agender (having no gender identity), genderqueer, or any other combination and self-label which indicates the individual has a gender identity that is not exclusively a man or a woman (Salinas-Quiroz and Demos-Utrera, personal communication, June 27, 2022).

Our goal is to begin to create a framework for understanding gender development in non-binary children ages 0 to 8. As there is virtually no empirical data on the subject, our research involved a thorough literature review of gender development theories, as well as knowledge gained from our own lived experiences as non-binary individuals. Using these methods and data, we postulate that there are two minimum criteria for non-binary gender identification: that a child learns about the existence of non-binary identities, and that they do not identify with the definitions they have been taught of what a boy or girl is.

To meet the first criterion for non-binary gender identification, parents, and other people with influence in a child's life must have the resources and desire to teach them gender as a spectrum.^1^ As discussed, activism by trans people of color has made huge strides in promoting awareness and understanding of non-binary identities. A 2015 Fusion Millennial poll showed that the majority of adults ages 18–34 in the US see gender as a spectrum (Wong, 2016). We will discuss how parents, peers and media may teach this to children.

Clearly not every young person who learns about non-binary identities will identify as such: the individual has to be taught the definitions of girl, boy and non-binary, and claim non-binary as their own. Definitions of gender contain a set of characteristics assigned to a label (i.e., boy, girl, non-binary). These characteristics include, but are not limited to, attitudes, behaviors, interests and physical presentation (Morgenroth and Ryan, 2018; Lindqvist et al., 2021), and for the purposes of this paper will be referred to as “gender traits.” Importantly, the definitions of gender labels that children learn are not always the same, and are largely contingent on the environments they grow up in. While they vary widely, there are two extremes within the continuum: environments with more liberal gender norms, and environments with more conservative gender norms. Liberal gender norms refer to broader and more expansive concepts of the gender traits associated with gender labels. Conservative gender norms refer to narrower and more rigid concepts of the gender traits associated with gender labels.

Both nature and nurture contribute to the development of gender traits. Biological factors influence predispositions toward or against certain characteristics. Parents and other adults encourage children toward or against certain activities, model gender traits and can destigmatize non-conformity. Lastly, the gender makeup of peer groups impacts the ability to explore gender traits freely. Environments conducive to gender trait exploration, allow children to realize their authentic selves, and for some, to identify as non-binary.

In 1960, the English pediatrician and psychoanalyst, Donald Winnicott, identified the true self as the authentic core of one's personality, from which spontaneous action and a sense of realness come (Ehrensaft, 2012). From in-depth interviews conducted with non-binary adults, non-binary professor, Waagen (2022), underlines the importance of living authentically to one's transgender identity, as it improves self-image and self-love. We borrowed the term *authentic* since it symbolizes the intersection of child and trans studies. Furthermore, contemporary authors in both fields conceive children as active players with agency in their development and not passive recipients and mere products of their nature and nurture (e.g., Amar, 2016; Gill-Peterson, 2018; Belsky et al., 2020). All in all, some aspects of who we are precede socialization and supersede biological sex (Serano, 2007). Both intrinsic and extrinsic factors help shape the way we come to experience and subsequently be cognizant of our authentic gender.

It is important to understand and create a framework for gender development of non-binary children instead of trying to fit them into theories designed for cisgender children; theories based on dated and narrow concepts of gender as well as science-disproven ideas of the sexually dimorphic brain in which non-binary identities represent an oxymoron. In this article we will discuss factors conducive to authentic gender identity development in non-binary children.

For didactic purposes our journey will be chronological, starting with prenatal biological development associated with “femaleness-maleness,” followed by socialization conducive to learning about non-binary identities and authentic gender development, first in young children ages zero to three, then ages four to eight. When it comes to understanding development, we believe that there is no nature without nurture (and vice versa). It is long past time to abandon this duality since we are dealing with systemically integrated human beings, not peoples with separate parts that follow entirely different rules (Belsky et al., 2020).

## 2. Biological contributors to gender trait development

The long-reigning theory on biological contributors to gender development states that prenatal exposure to testosterone causes “male-typical development” (i.e., masculinization), in as much as “female-typical development” (i.e., feminization) occurs in the relative absence of this hormone. Specifically, testes develop from the embryonic gonad under the influence of multiple genes that begins with the expression of the SRY on the Y chromosome. On the other hand, ovaries develop under the influence of a cascade of genes that are influenced by the expression of DAX1 on the X chromosome and act antagonistically to SRY (Wilhelm et al., 2007). Accordingly, it has been indicated that genitals' sexual differentiation takes place during the first trimester, while the sexual differentiation of the brain occurs in the second trimester. One possible explanation of the timeline has to do with the influence of sex hormones on the developing brain cells (Swaab, 2007; Roselli, 2018).

“…[W]hile animal studies provided plenty of evidence that testosterone affects multiple aspects of the brain structure, brain structure is also influenced, in both, males and females, by other sex-related hormones and by sex-related genes…This is expected to lead to higher variability in the “femaleness-maleness” of different features within a single brain that the one expected in the case of a single factor. Moreover, sex-related hormones, including testosterone, act on different brain features via multiple independent mechanisms, so that even features affected by the same hormone, may vary considerably in their location along their female-male continuum…” (Joel, 2021; p. 166).

In a nutshell, these views have proven to be overly simplistic, and based on the science-disproven idea of the sexually dimorphic brain (Joel et al., 2015), as well as overgeneralizations by researchers and misrepresentations stemming from complex language and confirmation bias (Joel and Fausto-Sterling, 2016). A slew of research in the last fifteen years has challenged these long-prevailing notions, citing technique flaws and inconsistencies, and providing evidence supporting an alternative theory: the mosaic brain [e.g., (Jordan-Young, 2010, 2012; Rippon et al., 2014, 2021)]. Although it is undeniable that there are sex/gender differences in brain and behaviors, humans and human brains are comprised of unique overlapping *mosaics* of male-typical and female-typical features, where internal consistency across characteristics within individuals is rare (e.g., Joel et al., 2015; Hyde et al., 2019).

Biological aspects of an individual absolutely have an impact on their gender development, however, it is much more complex than it has historically been made out to be. We laud the researchers disproving the harmful and inaccurate theories of binary sex, and currently do not believe the field is at a place to make general assertions of causation between biological structures and gender development with any conviction.

## 3. Gender socialization from ages 0 to 3

### 3.1. Learning about non-binary identities (between ages 0–3)

While many parents who hold more liberal gender norms acknowledge the importance of teaching gender as a spectrum, the majority have little understanding of what it is to be non-binary, or how to explain it. As a result, the primary source from which young children may learn about non-binary identities and the spectrum in gender is through books and other media [e.g., *They, She, He, Me* by Gonzalez and Smith-Gonzalez (2017), Terrace (2020-2023)]. Another way children learn about non-binary identities is through education by and contact with friends and family members that are part of the 2SPLGBTQIA+ community. These are some ways the first criterion for non-binary gender identification (learning about non-binary identities) can be met.

### 3.2. Authentic trait development (between ages 0–3)

The first 3 years of life are fundamental for gender socialization. During this critical period, most children are almost exclusively socialized by their parents. According to Quinn et al. (2002), children learn to differentiate between women and men at around 6 to 10 months, likely done on the basis of characteristics culturally linked to gender (Leinbach and Fagot, 1993). This is evidenced by their ability to differentiate between pictures and voices of men and women (Miller, 1983; Leinbach and Fagot, 1993). By the time babies turn one year old they start learning gender stereotypes (Levy and Haaf, 1994). This initial period influences to a great extent the filters children will have when presented with gender related material in the future. By their second birthday their behavior is not only influenced by the stereotypes, but they distort new gender-related input to fit them (Trautner et al., 2003; Martin and Ruble, 2004). It is reasonable to extrapolate that the more rigid the gender norms and definitions one has been taught, the more children will have to distort gender related information to fit them. However, when parents specifically undertake an effort to reduce the rigidity of gender socialization, it increases children's beliefs of the expansiveness of gender norms and definitions (Istar Lev, 2010). More expansive ideas about gender allow for greater freedom exploring different activities, forms of presentation and other gender traits, which support authentic gender development (Fiani and Han, 2018; Dowers et al., 2020; Waagen, 2022).

Parents also play a key role in the salience of certain interests in their children. Despite the commonly held assumption that biological factors play a unilateral role in certain gender traits, modeled enjoyment and parental prompting has shown to work in congruence with biology (Jacobs and Eccles, 1992; Jodl et al., 2001). For example, parents wanting their kid to play sports has shown to be a factor in boys' interest in the activity (something generally presumed to be “natural”), as well as influential in increasing girls' interest in sports. Encouraging children to explore their interests, particularly when in opposition of traditional gender norms, has shown to be comparably influential to biological pre-dispositions, and important to children feeling supported in their authentic selves.

## 4. Gender socialization from ages 4–8

### 4.1. Learning about non-binary identities (between ages 4–8)

While some children will have already learned about non-binary identities before starting school, it becomes increasingly likely that children will be taught about gender as a spectrum beginning in pre-k and elementary school. Some parents may not learn about non-binary identities until they start interacting with teachers and other parents. Other caregivers may choose to introduce the topic now that their child is older in many of the same ways previously mentioned. Further, if parents had chosen not to teach their child about non-binary identities, once children spend much of their time out of the house, it is more likely they will learn from another adult, community member or form of media.

### 4.2. Authentic trait development (between ages 4–8)

Imitating models is an influential factor in the development of gender traits, not only through receiving representation, but reinforcement and punishment as well. Children imitate behaviors in people with characteristics they identify with and place importance on (Blakemore et al., 2009), categorizing others but also themselves to recognize the shared traits. While there is a strong preference for the imitation of same-gender models in boys, there is significantly less of a same-gender bias in girls (Bussey and Bandura, 1984; Blakemore et al., 2009). This raises the question, who do non-binary kids imitate, and what models are important for them to have? While having some non-binary models would be ideal, non-binary children, just like girls, will find shared traits in people of different genders. This being said, it is incredibly important that non-binary children see their most stigmatized traits, characteristics of gender non-conformity, modeled. As non-binary author and social worker Koonce (2019) states from their personal and professional experience, given the lack of a non-binary gender definition “...it's in the mirroring of others that [non-binary identities] truly take form” (location 3,021), and research has demonstrated the importance of exposure to models with different gender presentations in encouraging ongoing exploration of gender traits (Kuper et al., 2018).

As children begin routinely spending extended time with same-age peers around the ages 3 to 5, they come into contact with many new activities, expressions, playstyles, and other gender traits. Further, Waagen (2022), discovered these ages to be a common time for non-binary people to recognize discomfort with the gender norms of their sex assigned at birth, prompting questioning of their gender identity. This exposure to new possibilities in conjunction with recognized cognitive dissonance is conducive to experimentation, however, the experience can be highly influenced by gender norms. Living in environments with rigid and conservative gender norms, for example a peer group that punishes gender-atypical behaviors (as previously discussed), act as a barrier to exploration (Fiani and Han, 2018; Kuper et al., 2018). On the other hand, individuals that perceive unconditional support from family and friends feel safer to explore new gender traits (Waagen, 2022). Environments with liberal gender norms are more conducive to authentic gender development in children (Fiani and Han, 2018; Dowers et al., 2020).

Peer groups contribute significantly to the confidence children feel to explore their interests and behaviors, and there are two main categories: single-gender groups, and mixed-gender groups. Throughout elementary school, single-gender groups are more common, exaggerate shared traits, and punish individuals who transgress gender norms (Fabes et al., 2003; Hanish and Fabes, 2014). This peer pressure may “greatly impede development or prevent authenticity to one's gender expression” (Waagen, 2022; p. 11). Experts affirm that peer groups form out of shared interests more than a preference for peers of a certain gender (Priess and Hyde, 2011). As non-binary children will likely have some preferences for gender traits atypical of their sex assigned at birth, they will spend much of their time in mixed-gender groups. The aforementioned involves “relatively non stereotyped activity choices” (Fabes et al., 2003; p. 930), as non-binary kids will find more support for their exploration and authenticity in these peer group settings.

## 5. Discussion

In order for a child to come to identify as non-binary, children need to learn about the existence of non-binary identities, and not identify with the definitions they have been taught of a boy or a girl. Children can learn about non-binary identities and gender as a spectrum through parents, media, and knowledgeable community members, and can develop gender traits authentically through biological predispositions, parental support, observational learning, as well as being in peer groups supportive of gender trait exploration.

It is imperative to dismantle adultcentric ideas that perpetuate the notion of children as passive recipients and mere products of their nature and nurture. It has been proven that children are active players with agency in their development (e.g., Amar, 2016; Gill-Peterson, 2018; Belsky et al., 2020). Being exposed to liberal gender norms will not “make them non-binary.” What seems plausible, however, is that those who do not see themselves represented, or do not feel respected in their social groups will actively seek models and friends who will validate their identity. All in all, “[non-binary folks] represent one idea and one idea only: how to be you” (Marsh, 2019, location 1,923).

Together with Jocelyn Demos-Utrera and Lucinda Garcia, fellow members of the “Abby and Anna” Sexual Orientation, Gender Identity and Expression (SOGIE) Lab, Eliot-Pearson Department of Child Study and Human Development at Tufts University, we have an ongoing project where we interview 3- to 8-year-old non-binary children and their parents.^2^ This study aims to investigate how these children understand their gender and gender-related experiences. Nine child-parent dyads generously agreed to participate and preliminary findings support the aforementioned criteria for authentic gender development: our children' participants have learned about non-binary identities, do not identify completely or exclusively as a boy or girl, and claim a non-binary identity, mainly through self-labeling and the use of “they/them” pronouns. All their parents hold liberal gender norms, self-educate on the gender spectrum, and show their children unconditional love and support for exploration. This article represents the first of a series where we seek to contribute to the deepening our understanding of non-binary identities to support these kids and honor the Brown and Black non-binary ancestors.

## Data availability statement

The original contributions presented in the study are included in the article/supplementary material, further inquiries can be directed to the corresponding author.

## Author contributions

FS-Q and NS contributed to the conception and design of the study, organized the literature review, and wrote the first draft of the manuscript. Both authors contributed to manuscript revision, read, and approved the submitted version.

## Footnotes

^1^For example, research has shown that teachers and other adults offer differential treatment (i.e., attention, type of response, instruction, and feedback) to people assigned male at birth (AMAB) and people assigned female at birth (AFAB). However, they are usually unaware of their biases and if recognized often believe that they are operating on fair assumptions based on the children's behavior (e.g., Sadker et al., 1994; Blakemore et al., 2009). We deliberately decided not to include information related to secondary adults in depth as the dominant literature suggest they generally reinforce cissupremacy and conformity to their assigned gender at birth and as a result it seems trivial for the postulated minimum criteria for nonbinary gender identification. Nonetheless, they are able to encourage authentic gender development in many of the same ways parents do.

^2^“How Do Children Identifying Beyond the Gender Binary and Their Parents Understand Gender?”. IRB ID: MOD-06-STUDY00002649. Tufts Social, Behavioral, and Educational Research Institutional Review Board.
